# Deservingness on the Front Lines: How Volunteers Navigate Moral Judgments in Emergency Food Distribution

**DOI:** 10.1111/cars.70011

**Published:** 2025-08-18

**Authors:** Carly Hamdon

**Affiliations:** ^1^ Department of Sociology University of British Columbia Vancouver Canada

## Abstract

The COVID‐19 pandemic marked a significant shift in the landscape of social assistance in Canada, as emergency support became more widely accessible. Faced with the prospect of rapidly distributing aid during an international crisis, this study draws on interviews with 19 volunteers from an emergency food program in Vancouver's Downtown Eastside to  explore how they determined which free food distribution strategies were appropriate. Findings show that decisions were shaped by cultural assumptions about deservingness and moral worth. Specifically, volunteers compared traditional food bank lineups, which were seen as stigmatizing and dehumazing, to the at home delivery service they implemented, which was considered more dignified. The decision to enact this free food distribution strategy also aligned with the neighborhood's ethos of social solidarity. By foregrounding how volunteers navigate moral judgments in their roles, this study contributes to broader sociological debates about the distribution of social assistance and the everyday moral labor involved in volunteer work.

## RÉSUMÉ

La pandémie de COVID‐19 a marqué un changement important dans le paysage de l'aide sociale au Canada, car l'aide d'urgence est devenue plus largement accessible. Face à la perspective de distribuer rapidement de l'aide pendant une crise internationale, cette étude s'appuie sur des entretiens avec 19 bénévoles d'un programme alimentaire d'urgence dans le quartier Downtown Eastside de Vancouver pour explorer comment ils ont déterminé quelles stratégies de distribution alimentaire gratuite étaient appropriées. Les résultats montrent que les décisions ont été influencées par des hypothèses culturelles sur le mérite et la valeur morale. Plus précisément, les bénévoles ont comparé les files d'attente traditionnelles des banques alimentaires, considérées comme stigmatisantes et humiliantes, au service de livraison à domicile qu'ils ont mis en place, jugé plus digne. La décision de mettre en œuvre cette stratégie de distribution alimentaire gratuite s'inscrivait également dans l'esprit de solidarité sociale du quartier. En mettant en avant la manière dont les bénévoles gèrent les jugements moraux dans le cadre de leurs fonctions, cette étude contribue à élargir le débat sociologique sur la distribution de l'aide sociale et le travail moral quotidien que suppose le bénévolat.

## 1 Introduction

During the initial phase of the COVID‐19 pandemic, food insecurity rates in Canada sharply increased (Statistics Canada 2020). By autumn 2020, however, these rates had declined to below pre‐pandemic levels, a change partly attributed to substantial social assistance spending by the Trudeau government during the crisis (Polsky and Garriguet 2022). In the first 8 months alone, the federal government invested $240 billion in social services, including funds for the Canadian Emergency Response Benefit (CERB) and the Emergency Food Security Fund, which disbursed $300 million to support food banks and other aid organizations (Gatehouse 2020; Report 12: Protecting Canada's Food System 2021). The expansion in federal aid during the pandemic was unexpected considering the historical trend in wealthy liberal democracies toward reduced social services and more exclusionary eligibility criteria for aid (Bloemraad et al. 2019; Dobrowolsky 2007).

I interviewed volunteers associated with a federally funded emergency food program in Vancouver's Downtown Eastside (DTES) during the COVID‐19 pandemic to understand how aid distributors grapple with cultural assumptions about need, and how these assumptions help determine who is eligible for aid. The central research questions guiding this project being: *How do volunteers decide which food distribution strategies are appropriate?* And *how do these decisions reveal underlying cultural assumptions about deservingness and morality?* My findings show how volunteers draw on observations of local food bank practices to determine which food distribution strategies are appropriate. More specifically, those I interviewed criticized approaches like public food bank lineups, perceiving them as harmful to the dignity and self‐worth of free food recipients. This critical engagement led volunteers to implement a door‐to‐door delivery service during the height of the COVID‐19 pandemic. These findings suggest that when volunteers work outside bureaucratized, charitable food banks and do not have to face the “moral dilemma” of deciding who is “deserving” and “undeserving” of aid (Maestri and Monforte 2020), they are able to enact practices that respect the dignity and moral worth of all free food recipients.

## 2 Background

### 2.1 Food Banks and Insecurity

An important area of sociological research is the issue of household food insecurity in rich, liberal democracies like Canada. Household food insecurity is conceptualized as inadequate or insecure access to nutritionally adequate and safe foods due to financial constraints (Reynolds and Mirosa 2018; St‐Germain and Tarasuk 2017). The most recent national estimate shows that 22.9% of Canadians live in a food‐insecure household, the highest rate in almost 20 years of monitoring (PROOF 2024). This is a significant public health issue. Even marginal (or low) rates of food insecurity are correlated with severely negative health outcomes—particularly in children (Gundersen and Ziliak 2015). Researchers continually interrogate the geographic, sociodemographic, and economic determinates of food insecurity in Canada, seeking to explain the paradox of “want amidst plenty” (Reynolds and Mirosa 2018).

Across Canada, food is readily available yet financially inaccessible for many individuals. The Household Food Security Survey Module (HFSSM) is a vital tool elucidating the myriad correlates of food insecurity across the country. The HFSSM is an 18‐item, experience‐based questionnaire first developed by the United States Department of Agriculture (St‐Germain et al. 2019) used to measure food access problems caused by a lack of money (St‐Germain and Tarasuk 2020). The inclusion of this unidimensional scale into population‐level studies in Canada reveals that the probability of experiencing food insecurity is affected by the household's provincial location (Tarasuk et al. 2014), highest level of education attained (St‐Germain and Tarasuk 2020, 2017; Tarasuk, St‐Germain, and Loopstra 2019), type of relationships between the people who live in a household together (St‐Germain and Tarasuk 2020), the presence of children in the household (St‐Germain and Tarasuk 2017), homeownership status (St‐Germain and Tarasuk 2020), and ‘Aboriginal Status’ (Tarasuk, St‐Germain, and Mitchell 2019; Willows et al. 2009). Strikingly, analysis of the 2008–2013 First Nations Food, Nutrition and Environment Study (FNFNES) reveals that 46% of self‐identified First Nations living on reserve experience food insecurity (Domingo et al. 2021).

Employing indexes like the HFSSM and FNFNRS in representative survey research reveals the most significant correlates between food insecurity and other social factors. It also emphasizes the need for a more fulsome social safety net (cf. Loopstra and Tarasuk 2012; Riches 2018). However, the predominate response to hunger in Canada is food banks, a solution that is currently “failing to address the structural causes of food insecurity” (Miewald and McCann 2014, 545).

Due to policy reforms that weakened the provision of income assistance and other publicly funded supports during the economic recession of the 1980s, rates of food insecurity in Canada rose dramatically (Loopstra and Tarasuk 2012). In response to this social problem, food banking grew rapidly (Tarasuk, St‐Germain, and Loopstra 2019). Food banks are extra‐governmental programs that distribute grocery‐type foods free of charge by relying on donations and volunteer labor (Loopstra and Tarasuk 2014; Tarasuk, St‐Germain, and Loopstra 2019). Despite food banks being originally implemented as a temporary solution to a crisis, they have become entrenched in Canada, at least in part due to the tax credits that corporations receive in return for donations (Tarasuk, St‐Germain, and Loopstra 2019). Today, Food Banks Canada includes 10 provincial associations and more than 5100 affiliate community centers (Food Banks Canada 2024). In spite of the proliferation of food banks across the country, studies show that visiting a food bank does not ameliorate food insecurity in the long term (Black and Seto 2018; Holmes et al. 2019). Rather, those who are food insecure tend to be long‐time users of free food services, highlighting how food banks work as a stop‐gap measure but not a structural solution. In addition to being an inadequate solution to the issue of food insecurity, the experience of receiving food from a food bank is often a negative one.

### 2.2 Food Bank Stigma

Relying on food banks for access to free food is often a socio‐psychologically damaging experience (Bruckner et al. 2021; Garthwaite 2016; Holmes et al. 2018; Middleton et al. 2018; Miewald and McCann 2014; Van der Horst et al. 2014). Feelings of shame, embarrassment, degradation, humiliation, awkwardness, failure, desolation, intimidation, guilt, discomfort, powerlessness, inequity, nervousness, and frustration are frequently expressed by free food recipients. These feelings are associated with specific free food distribution strategies. For example, interviews with free food recipients reveal that receiving poor quality (and expired) food causes decreased feelings of self‐worth (Enns et al. 2020; Middleton et al. 2018; Miewald and McCann 2014; Van der Horst et al. 2014), while having to wait in public lineups to receive free food is associated with feelings of humiliation, shame, and mental exhaustion (Bruckner et al. 2021; Douglas et al. 2015; Enns et al. 2020; Miewald and McCann 2014). These negative emotional experiences are both external (inflicted upon recipients) and part of their “inner theatre” (as they react to the perceptions of others) (Scheff 2000). In other words, shame is the individual‐level manifestation of “poverty stigma,” or the assumption that low‐status groups lack self‐sufficiency, ambition, and a sense of responsibility (Lamont 2019; Reutter et al. 2009). The stigma surrounding free food not only negatively shapes the experience of those in need but can also inhibit individuals from accessing available services (Bowe et al. 2019; Dodd and Nelson 2020; Garthwaite 2016), limiting the efficacy of an already limited solution.

Preliminary research suggests practices which may aid in the destigmatization of those who rely on access to free food and improve their experiences. For instance, qualitative research suggests providing high‐quality food (which recipients have some choice in selecting), changing the way that food parcels are provided to recipients (by eliminating lineups), and encouraging more community building (where volunteers and recipients may interact and participate in decision‐making) to improve experiences of receiving free food (Enns et al. 2020; Middleton et al. 2018; Van der Horst et al. 2014; Vissing et al. 2017). In fact, implementing novel approaches, such as choice models of food selection and onsite programming such as nutrition education, life‐skills training and social support services, is associated with slight improvements in perceived mental health among free food recipients (Rizvi et al. 2021). In this way, destigmatization not only requires increasing material resources to low‐status groups. It also requires that low‐status individuals and groups gain recognition, validation, and membership into wider culture (Clair et al. 2016; Lamont 2018, 2019; Lamont and Mizrachi 2012).

### 2.3 Deservingness Judgements

Distributing poor‐quality food via public lineups reinforces poverty stigma and the belief that free food recipients only deserve the bare minimum. Prior research suggests that practices which make a difference in the lives of people who rely on access to free food are those which reinforce social similarities between groups and narratives that avoid blaming the low‐status group for their current predicament (Clair et al. 2016; Garthwaite 2016, 2017). In this way, the process of destigmatization is partially material, relying on the implementation of dignifying practices, and partially cultural, relying on a “plurality of criteria of worth” (Lamont 2019) or the shared idea that all people are worthy of social support regardless of socioeconomic status or position.

The cultural dimension of destigmatization requires individuals, particularly those who decide who is and is not “deserving” of aid, to grapple with and challenge pre‐existing stereotypes (Lamont and Mizrachi 2012). This becomes challenging in situations where there are inadequate resources; that is, not everyone in need will be able to access the money, food, or shelter they need to survive. For instance, volunteers assisting refugees in the United Kingdom and France face crippling “emotional and moral dilemmas” in their daily work (Maestri and Monforte 2020). To cope with the burden of deciding who deserves aid, volunteers construct representations of refugees. These representations then reinforce divisions between “the desirable” (the suffering and helpless) and “the undesirable” (the potentially threatening) refugee. The constant pressure to make these difficult decisions also leads to volunteer burn‐out. To cope, volunteers engage in other strategies—such as avoiding the responsibility of deciding who is (not) deserving of assistance altogether. “Moral outsourcing” occurs when volunteers shift the responsibility of making deservingness judgments to charity or the government (Garthwaite 2017; May et al. 2019; see also Maestri and Monforte 2020). This practice allows volunteers to avoid reckoning with emotional dilemmas and to take a more comfortable, non‐judgmental stance.

The volunteers I interviewed in this research project did not engage in moral outsourcing. Instead, the volunteers I interviewed made deservingness judgements throughout the entirety of the free food program—a process made less burdensome by the ample funding provided by the Canadian government at the time. As such, this research presents a novel case to the literature on volunteer decision‐making processes, highlighting the possibilities of social action in extraordinary times. In many ways, this exceptional case is owed to the unique history and legacy of the neighborhood it is situated within—Vancouver's DTES.

## 3 The Case

### 3.1 The DTES

**FIGURE 1:**
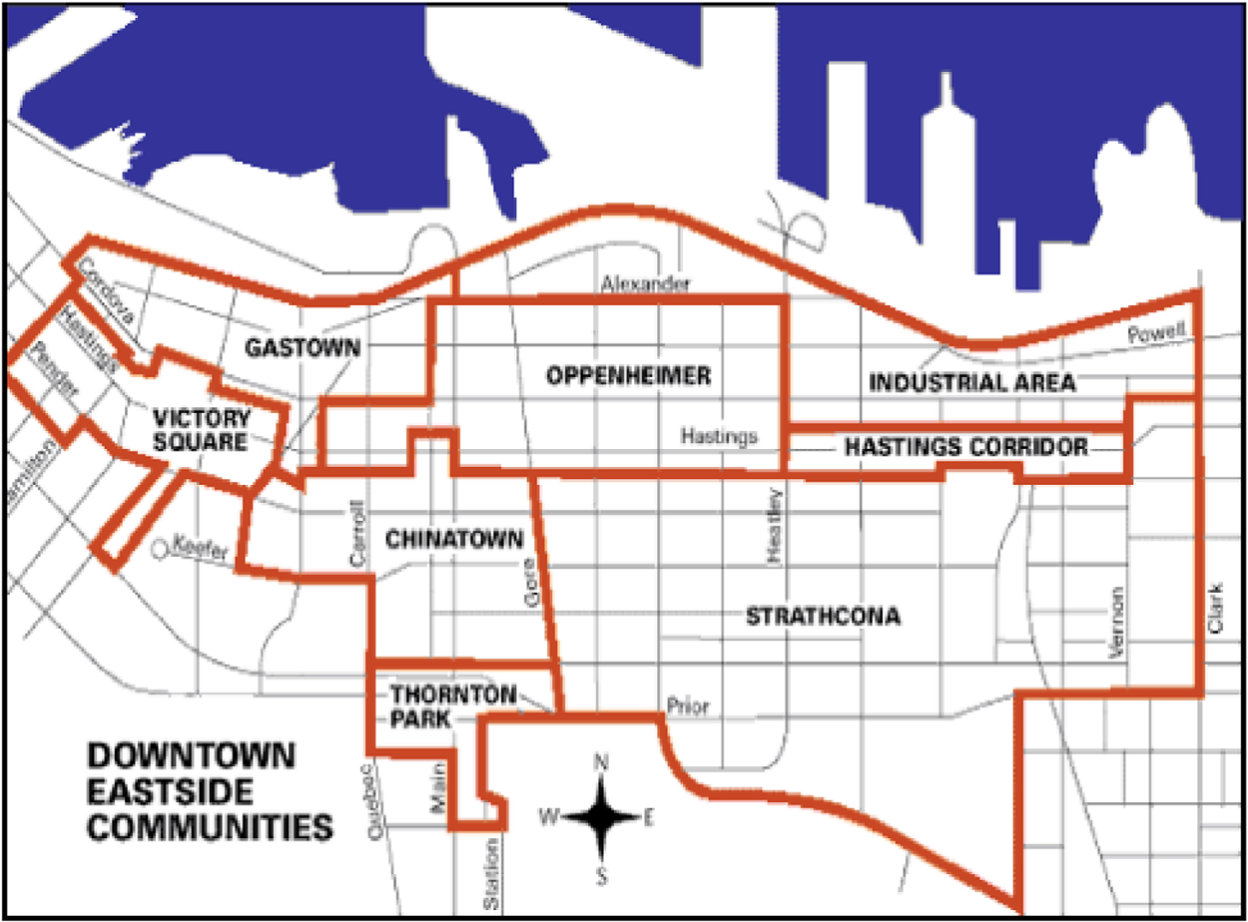
The Downtown Eastside (Newnham 2005). [Colour figure can be viewed at wileyonlinelibrary.com]

My participants volunteered with a community food security initiative located within the complex cultural landscape of Vancouver's DTES (Figure 1). The city of Vancouver was born in the DTES (Roe 2010) when the area was taken from the Stó:Lo people and transformed into a residential area for loggers and fishers (Newnham 2005). A transient, predominately male workforce who needed affordable accommodations, Japanese Canadians built an abundance of single‐room occupancy hotels and rooming houses in the mid‐19th century for the European and Scandinavian working poor (Drabble 2015; Roe 2010; Sommers 2001). Towards the end of the 19th century, many of the European men and their families could afford to move away from the DTES, and the neighborhood became rapidly demarcated as a non‐white, unsafe space (Newnham 2005). In addition to the “White Flight,” the ready availability of drugs and prostitution due to the transient male workforce contributed to the neighborhood's stigmatized identity (Sommers 2001). This time was also characterized by lingering racial tensions; for example, two anti‐Chinese riots took place in Vancouver's Chinatown in 1887 and 1907 (Guo and Guo 2021). Given these factors and the disappearances and murders of 69 sex workers between 1970 and 2002 (Robertson 2007), the DTES continues to be associated with risk (Sommers 2001). Today, the DTES is characterized by extremely high rates of drug use, poverty, crime, infectious disease, and mental illness (Linden et al. 2013). Many residents of the DTES experience severe food insecurity and rely on access to free food daily (Miewald and McCann 2014).

Though the neighborhood has been pejoratively called “skid row” (Robertson 2007), there is also a long history of activism and federally funded social assistance in the DTES. Notably, *Insite*, Canada's first safe‐injection site, is in the DTES. Additionally, urban geographers have noted that “food is everywhere” in the DTES, with “soup kitchens, community gardens, corner stores and trendy cafes” decorating the urban landscape (Miewald and McCann 2014, 538). The majority of the 100 charitable food resources in Vancouver are located in the DTES (Drabble 2015), and the neighborhood also houses a plethora of Indigenous‐focused and ‐led support services (Reporting in Indigenous Communities 2022), including Indigenous food sovereignty initiatives (Morrison 2008). The “insider‐versus‐outsider” tension over who knows what is best for the DTES (Roe 2010) contours the complicated neighborhood my participants are situated within.

### 3.2 Organizational Affiliation and Structure

The singular community food organization I drew my participants from is nested within the Vancouver Neighborhood Food Network (VNFN) and Community Food Centres Canada (CFCC). The VNFN is a coalition of citizens, organizations, and neighborhoods which work collaboratively to improve access to healthy, affordable, and nutritious food (Vancouver Neighbourhood Food Networks 2024). In action, the VNFN facilitates connections between otherwise disparate community food organizations and ensures that each neighborhood in metro Vancouver has a center committed to bolstering food access. CFCC works at a larger scale. CFCC is a national non‐profit that supports the broader food movement across Canada, facilitating knowledge sharing, creating health‐focused programs, and advocating for equitable food policy (Community Food Centres Canada 2024). Additionally, CFCC builds food centers in low‐income communities. Though separate, the VNFN and CFCC are united in their desire to reduce food insecurity, bolster food education, and build community. Both organizations also straddle the line between “progressive” and “reformist” approaches (Holt‐Giménez and Wang 2011; Poppendieck 2022), implementing traditional food bank model type‐programs that seek to aid immediate food insecurity as well as food justice programming and Indigenous food sovereignty initiatives.^1^

Established more than two decades ago, the local organization my participants are situated within is fraught with the same tension as its parent organizations. On the one hand, the organization is devoted to grassroots administrative principles and seeks to include all community members in the implementation of food programs. These programs include school gardening initiatives, bulk buying programs, community potlucks, and food literacy programs.^2^ On the other hand, the organization I drew my participants from implemented a federally funded emergency food program in response to an increase in food insecurity in the DTES during COVID‐19. While these divergent approaches appear irreconcilable, they are symptomatic of a larger, cross‐national food movement best understood as “a movement of movements” (Wilson and Levkoe 2022), which gains strength from inter‐movement allyship and alliance.

### 3.3 Community Entry

Days after the World Health Organization declared COVID‐19 a global pandemic in March of 2020, Vancouver food banks temporarily closed their doors. Overwhelmed by the immediate need for free food in the DTES, the lead organizer secured available federal funding and used it to purchase food (mostly produce) in bulk, though some was donated by local businesses in the area. The food was distributed to those in need in customized boxes. The emergency food program began in March of 2020 and ran for 18 months, distributing thousands of pounds of food to individuals and families in the DTES. I volunteered as a box organizer and delivery driver throughout the winter and spring of 2021. Volunteering allowed me to develop a trusting relationship with the lead organizer and helped inform my interview guide. For example, while packing boxes, I observed how free food recipients’ dietary concerns and cultural preferences were considered. Additionally, locally caught salmon was often set aside from recipients who identified as Indigenous and requested culturally appropriate food.

Because group context has a significant impact on the structure and logic of political discussion (Perrin 2005), the community food organization I drew interview participants from should be considered as a critical case; one nested within a geographic context rife with social problems and solutions. By laying out an abridged history of the DTES, the structure of the community food organization, and the program they ran during the COVID‐19 pandemic, I sought to highlight how those who live, work, and volunteer in the DTES are situated in a legacy of dispossession and activism, of crisis and hope. To spend time in the DTES is to bear witness to systemic problems and a plethora of methods used to address them.

## 4 Methods

### 4.1 Data Collection and Analysis

The data for this study included 18 semi‐structured, in‐depth interviews with 19 participants, all of whom were involved with an emergency food delivery program on Vancouver's DTES.^3^ Given my connection with the organization, snowball sampling was an effective way for me to recruit participants. I circulated research invitations at a volunteer appreciation Zoom event and through email. I conducted interviews over Zoom from July to August 2021. While internet platforms such as Zoom can create moments of disjuncture between interviewer and interviewee, potentially disrupting emotional connection and creating an unpleasant “affective atmosphere” (Adams‐Hutcheson and Longhurst 2017), videoconferencing provides many of the same advantages of traditional in‐person interviews (Irani 2019) with the additional benefits of increased convenience, flexibility, and safety for both researchers and participants.

Interviews ranged from 25 to 90 minutes and produced 239 pages of single‐spaced textual data. To analyze my data, I engaged in a thorough process of thematic analysis in NVivo (Clarke and Braun 2017; Ryan and Bernard 2003). Unbounded by theoretical commitments, thematic analysis of qualitative data begins with rounds of open coding, where all data that is potentially relevant to the research question is identified. An initial round of open coding resulted in 71 inductive codes. I then used the constant comparative method, systematically comparing each text assigned to a category with each of those already assigned to that category (Glaser 1965; Zhang and Wildemuth 2009). This helped eliminate redundant or irrelevant codes and improved the reliability of the remaining codes as the central properties of each one was clarified. Finally, I engaged in several rounds of axial coding in which I grouped my open codes into three larger themes (see Table 1) (Corbin and Strauss 2008). These themes provided an answer to my research questions, and structure the findings sections of this research article.

**TABLE 1:** Key themes and associated codes.

Themes	Associated codes
Solidarity	Quarantine; Mobility; Childcare; Layoff; Vulnerability; Isolated; Facebook; “the Community”
Stigma	Shame; Embarrassment; Food Bank; Line Up; Physical Access; No Choice (of Food); Poor Quality (Food)
Dignity	Food Delivery; Food Choice; Culturally Appropriate Foods; Respect; Anonymous; Healthy Food; Connection

### 4.2 Participant Demographics

The selection criteria for participants included anyone who helped organize or distribute food for the community food organization's emergency food program during the COVID‐19 pandemic. Unlike much existing food literature, which explores poverty stigma by talking to food bank users (Enns et al. 2020; Holmes et al. 2019; Middleton et al. 2018; Miewald and McCann 2014; Millar et al. 2020), my sample of volunteers and organizers presents a unique case study. By interviewing committee members, organizational leaders, and volunteers, this research highlights how those tasked with the responsibility of allocating social service provisions recognize and disrupt otherwise stigmatizing practices. Regarding participant demographics, 13 of my research participants (or 68% of my sample) self‐identified as “white,” “Caucasian,” or of solely European descent (see Table 2). The racial demographics of my sample reflect the whiteness of volunteer organizations in the Western world (Maestri and Monforte 2020) but not the Greater Vancouver area, a region where 55% of residents are classified as “visible minorities” (Statistics Canada 2021). As such, the racial identity of my sample is not representative of recent population demographics for Vancouver. However, representativeness was not a central goal of this study. Rather, I needed to speak to people to whom food inaccessibility *mattered* (cf. Swidler 2001 for a similar approach). Arguably, those who risked their health and spent their time participating in an emergency food program through the height of the COVID‐19 pandemic care deeply about this issue.

**TABLE 2:** Participant demographics.

Sociodemographic variables	*N* = 19
**Age**	
25–34	3
35–44	2
45–54	3
55–64	9
65–74	2
**Gender**	
Male	5
Female	14
**Employment status**	
Employed full time (40 or more hours a week)	5
Employed part time (up to 39 hours a week)	4
Self‐employed	3
Student	2
Retired	5
**Race/Ethnicity**	
White/Caucasian/Entirely European Descent	13
Chinese	3
Indian	1
Iranian	1
Southern‐American	1
**Highest level of education**	
Less than high school	1
High school	0
Some college, no degree	3
Bachelor's degree	9
Master's degree	5
**Estimated household income**	
$25,000–$50,000	7
$50,000–$100,000	3
$100,000–$150,000	3
$150,000–$200,000	2
$200,000 or above	2

## 5 Findings

At the onset of the COVID‐19 pandemic, social assistance became widely accessible to residents across Canada. However, volunteers operating an emergency food program in Vancouver's DTES faced difficult questions about who deserved free food (as resouces were ultimately not limitless) and how it should be distributed. This study addresses two central research questions: *How do volunteers decide which food distribution strategies are appropriate?* And *how do these decisions reveal underlying cultural assumptions about deservingness and morality?* The answer is threefold. First, volunteers emphasized their deep connection to the DTES, expressing a strong sense of social solidarity. Second, they described public, outdoor food bank lineups as inappropriate, viewing them as stigmatizing and undignified. Third, they supported the program's alternative delivery model, which they saw as a more respectful and community‐oriented approach. Together, these findings show how volunteers assumed responsibility for determining both who deserved free food and how it should be provided—working to preserve the dignity and privacy of community members during a time of crisis.

### 5.1 Solidarity

As many might remember, the COVID‐19 pandemic and subsequent quarantine measures limited many people's physical and financial access to food. The volunteers I interviewed were quick to point out how many individuals in the DTES were “quarantining or having mobility issues” (Barry, delivery driver), making it difficult for them to access food. Other participants added that seniors, those with disabilities, and people with children also struggled to get to food outlets. Several participants also noted that the onset of the pandemic resulted in a wave of workplace closures and layoffs. The impacts of these layoffs were made visible through a neighborhood Facebook group. In this group, people posted that they suddenly could not pay for food or afford rent. To Chelsea (delivery driver), it was “horrifying to see that, just in my own community, this was happening. And, it was so rampant. There were hundreds of posts a day sometimes.” Critically, the horror volunteers felt in response to the suffering around them led not to inaction but to decisive engagement. As they recognized the issues their neighbors faced, they took initiative—volunteering their time and energy to the organization they believed was the most community centric.

If two words capture the lead organizer's core values, they are “community building.” Mathew explained that throughout his life as a community organizer, his goal has been to strengthen the resiliency of those around him. He spoke fondly about working in the DTES, saying it is a place where “there is that sense of community.” He elaborated: “people come out and support you” when you need it, and “we take care of each other, we support each other.” As wave after wave of people in the DTES expressed their struggles on a local Facebook page, those I interviewed told me how their decision to volunteer for the community food organization was because of its attention to community building and mutual aid. For instance, Charlotte (delivery driver) was motivated to volunteer because the emergency food program did not appear to be designed by “someone else,” rather, “it was people within the community volunteering and essentially helping their neighbors.” Similarly, Chelsea got involved because she wanted “to help people in my community” and perceived the emergency food program as an opportunity to do so.

As detailed above, Vancouver's DTES is a unique neighborhood characterized by its strong sense of social solidarity (cf. Miewald and McCann 2014). Volunteers emphasized the struggles facing community members and expressed their desire to work with an organization rooted in the DTES. Moreover, when volunteers began organizing the emergency food program, they shared a core belief: no one should go hungry, and helping those in need was simply the right thing to do. The solidarity experienced by volunteers became a guiding force for their efforts and helps explain why they found food bank lineups an especially troubling practice.

### 5.2 Stigma

Driving by a local food bank lineup, Bonnie (committee member) recalled thinking: “there's no cover. There is no shelter. There's a visibility there, it marginalizes people.” When interviewing those involved in an emergency food program in Vancouver's DTES, volunteers (including committee members like Bonnie) would often draw stark comparisons between their program and typical food bank operations. One free food distribution strategy that volunteers associated with food banks were public, outdoor lineups. As evinced by Bonnie, lineups are seen as socially uncomfortable. Some volunteers perceived line ups as stigmatizing. For example, Charlotte thought that “sometimes there can be a stigma with that, the lining up for food” as they make one's need for social assistance public. Indeed, research on food bank lineups emphasize how they separate and stigmatize those in need (Bruckner et al. 2021; Miewald and McCann 2014; Van der Horst et al. 2014). In addition to being socially uncomfortable, the volunteers also emphasized how physically uncomfortable standing in an outdoor, public line up often is.

“If you go to the food bank in Vancouver,” explained Chelsea, “you will see a line of going down the block for food… And you're having people who are disabled, elderly, stand outside in a lineup in the hot sun on concrete… [which] is not good for their joints.” Similarly, volunteers recalled witnessing “people… standing on the street in the heat or cold for hours at a time” (Crystal, committee member). Rather than pity these individuals, my participants expressed empathy towards them. For example, Valerie (committee member) recalled

hearing about seniors and folks with disabilities who were waiting two, three, four hours in line to get groceries in the middle of the pandemic back when it was super cold and rainy and snowy. Hearing about that, it made me cry. It made me so angry, because I just kept thinking – what would have happened had we also been in need?

Like Bonnie, Valerie disapproved of uncomfortable food bank lineups. However, she goes a step further by imagining herself in the same situation as people experiencing food insecurity. Valerie knows that she does not deserve such treatment; therefore, others do not deserve that treatment either. This sincere expression of empathy, elsewhere referred to as “drawing equivalences,” is a mechanism of cultural destigmatization (Clair et al. 2016). These empathetic expressions were common among the volunteers, three of whom cried during our interviews.

While a few volunteers (*N* = 2) believed the food distributed by traditional food banks was of particularly low quality, most were more concerned with the practice of public lineups. They unanimously agreed that lineups were an inappropriate method for distributing free food. Volunteers described these lineups as both stigmatizing—because they exposed people in moments of vulnerability—and dehumanizing—because recipients had to endure physical discomfort to access something as fundamental as food. From the volunteers’ perspective, such lineups were morally troubling and violated the sense of solidarity that defines the DTES community. In contrast, they saw personalized deliveries as a more dignified and appropriate way to provide free food.

### 5.3 Dignity

Rather than implement a food‐bank style lineup, the emergency food program delivered food directly to recipients’ homes. Perhaps unsurprisingly, my participants fully supported this method. For instance, Babette (delivery driver) reflected on the idea that,

this program probably had a lot less stigma attached to it. You were getting a friendly delivery from someone that shows up and they give it to you… and your neighbors don't know it's a food program. Right? It could be any food delivery.

With food delivery services more popular than ever during the COVID‐19 pandemic (Hong et al. 2021; Jia et al. 2021), participants like Babette perceived the free food boxes as inconspicuous. And, because the boxes did not seem to be obviously associated with food banks or free food, volunteers perceived them to be a superior method. This sentiment was echoed by several other participants. While there is often a “stigma around having to ask for food or to receive food,” David (delivery driver) sensed that “there was never any stigma or shame [with the food delivery].” In fact, he laughed boisterously as he told me about the “platonic love affair” that had sprung up between himself and one of the recipients. This recipient was a man far older than David, but they bonded over their shared Italian heritage and built a lasting friendship. By delivering food to recipients’ homes, interactions between individuals with different class backgrounds occur in (semi) private spaces, or “zones of encounter” (Miewald and McCann 2014 551). In these (semi) private zones, individuals are protected from the assumptions of outsiders and afforded the possibility to forge understanding and connection, a possibility my participants valued deeply.

In contrast to the food bank, which distributes food that the volunteers claimed they would never eat, the emergency food program allowed recipients to “choose what they want” (Chantel, delivery driver). Remarkably, the lead organizer personally called community members to inquire if they wanted (not needed) a food box and if they had any dietary restrictions or preferences—a practice fully supported by the volunteers. Rather than engaging in moral outsourcing, the organizers and volunteers took full responsibility for decision‐making processes. Chantel explained that the lead organizer (and program at large) was “very respectful of [recipient's] culture in terms of what they sent in the boxes.” Rather than make assumptions, participants noted that the lead organizer strove to treat free food recipients fairly and with equity. In fact, the program distributed grocery gift cards once a month, a practice that many volunteers, including Chantel, “thought was incredible.”

As discussed, the lack of choice that free food recipients experience at typical food banks perpetuates stigma and often manifests as internalized shame (Bruckner et al. 2021; Miewald and McCann 2014; Van der Horst et al. 2014). The emergency food program my participants were involved in sought to counteract these stigmatizing procedures, implementing strategies that were more private and ensure recipients were “treated with some level of dignity” (Chelsea). These strategies reflect and contribute to the volunteers’ broad criteria of deservingness; not only do all community members deserve free food, but they also deserve to exercise agency over the food they receive. Finally, my participants supported the actions taken by the lead organizer and committee members to secure fresh produce and high‐quality food for the recipients.

In their description of the emergency food program, my participants consistently emphasized how the boxes were “very healthy” (Babette) and included items such as “leafy vegetables, fresh vegetables, seasonal vegetables” (Delilah, delivery driver) which were of “superb” quality (Scott, delivery driver). In fact, one participant criticized the program for having food standards that were too high, therefore contributing to unnecessary food waste (Gina, box organizer). Another delivery driver, Charlie, recalled getting emotional over the food quality the emergency food program distributed. During a delivery, she recalled

looking at these baskets of food that were — and it's beautiful food. It is really good quality food and prepared soups. All the food was… tailored to the family what they needed, what they ate. So, you weren't just getting “Here, everybody gets a loaf of shitty bread and some peanut butter and some bruised apples and away you go,” it was beautiful food. And there were a couple times I almost wept when I looked at the food. It's like, ‘Oh my God, I can't believe this.’

Charlie was moved by the dignity with which the organizers treated the free food recipients. Not only did the emergency food program facilitate recipient food choice, but it committed to distributing high‐quality food—food that participants would eat, too. Altogether, these strategies treat recipients as worthy, valuable individuals who deserve care and respect.

During my interviews with the leader, committee members, and volunteers of an emergency food program on Vancouver's DTES, participants frequently deliberated on distinct strategies for distributing free food, drawing comparisons between their food program and the food bank. Most often, they critiqued the food bank and praised their program. Specifically, volunteers thought that food bank lineups stigmatized recipients and that their program protected the privacy and dignity of those reliant on free food. Moreover, they contrasted the perceived low quality of food offered by the food bank, where recipients have no say over what they receive, with the care and attention their leader invested in catering to recipients' needs. While discussing specific strategies of free food distribution could be misconstrued as superficial, these discussions revealed broader cultural assumptions—like the need to protect the moral worth of those in need of free food. In this way, the narratives of those working within the emergency food program during COVID‐19 demonstrated a “plurality of criteria of worth” (Lamont 2019), rejecting the idea that some people are “winners” while others are “losers.”

## 6 Conclusion

Deservingness judgements determine who recieves social assistance and who does not. This study provides a unique snap shot into a moment when government funding was bountiful, and those involved in the implementation of a social assistance program had the liberty of deciding which free food distribution strategies to employ. The volunteers I spoke to found food bank lineups dehumanizing and personalized deliveries anonymous and kind. In this way, the strategies deemed correct by volunteers were informed by a sense of social solidarity and a strong desire to do what was right—that is, treat all people with dignity and respect. These findings highlight the moral work that these volunteers engaged in throughout the duration of the emergency food program. There are practical and theoretical implications of these novel research findings.

My research participants’ efforts to distribute available resources to neighbors in need demonstrates how “power matters, but so does paying attention to normative claims and cultural scripts” (Bloemraad et al. 2019, 96). For instance, the idea of those in “the community” suffering due to COVID‐19 restrictions motivated volunteers to act. Moreover, witnessing food bank lineups and seeing oneself in the eyes of another influenced their decision to protect the anonymity of free food recipients. In this way, localized cultural scripts surrounding community solidarity were essential to the organization of the program, influencing how material resources were allocated. Moreover, these localized norms contributed to the volunteer's sense of morality. While previous research shows how volunteers avoid making decisions about deservingness (Garthwaite 2017; Maestri and Monforte 2020), the volunteers I interviewed leaned in, passionately advocating for certain practices they felt were the correct way of doing things. As the emergency food program only operated for a year and a half, future research should explore how the amount of time spent volunteering influences the degree to which volunteers engage in moral outsourcing.

The volunteers’ experiences witnessing food bank lineups, volunteering with other local or community food programs, and receiving free food themselves, helped them decide who deserved food and how that food should be distributed. As such, volunteers meaningfully engage with the world around them, discussing and pursuing social change through the pathways available to them (Gamson 1992). Moreover, the volunteers’ “small‐p politics” (Kennedy et al. 2017), or the convivial and pragmatic strategies of action my volunteers pursued, demonstrate how people involved in food movements seek to effect change through everyday social practices. Together, these findings build on previous research stressing the potential of localized food movements to effect change (Andrée et al. 2018; Nelson et al. 2018; Rosol et al. 2022). However, this research is limited in its ability to assess the degree to which free food recipients felt that they were respected or destigmatized. Future research will benefit from an increased sample size and the inclusion of recipients as well as volunteers.

Finally, recent studies on stigma deflection found that the mitigation of stigma for one group may result in the retrenchment of stigma for an equivalent group (Phung et al. 2021). My participants often compared the emergency food program's strategies to food bank strategies, endorsing the former and criticizing the latter. Future research should interrogate the possibility of stigma deflection within and between free food distribution programs, especially when both are located within a stigmatized neighborhood.

This paper has examined how volunteers navigate and enact moral logics of deservingness in routine encounters with free food recipients, revealing that deservingness is not simply imposed from above but is actively interpreted, contested, and negotiated through everyday encounters. By analyzing an emergency food program on Vancouver's DTES, my findings show that volunteers draw on a deep sense of social solidarity to guide their decision‐making processes—embracing the decision to operate a personalized food delivery service. Beyond feelings of solidarity, discussions of free food distribution strategies reveal volunteers’ diverse moral repertoires—including notions of vulnerability, dignity, respect, and care. These moral evaluations shape how resources are distributed, how recipients are treated, and how policy goals are translated into practice. Ultimately, this approach re‐centers the role of frontline volunteers in the moral politics of the welfare state, highlighting both their discretionary power and their entanglement in broader structures of inequality and governance.

## Endnotes

1 Both collectives are attentive to the ways in which settler‐colonialism continues to disproportionately affect Indigenous peoples. The VNFN is committed to decolonizing food practices on Unceded Coast Salish Territories, while CFCC has piloted programs such as the “Mind Your Food” initiative which designed to improve the wellbeing of young people in Indigenous, newcomer, and lower‐income communities.

2 Food literacy is defined as “personal behaviors for planning, selecting, preparing, and eating healthy foods [that] are considered a necessary life skill” (Begley et al. 2019, 14).

3 All interviews were conducted one‐on‐one except a single interview where I interviewed a couple jointly.
